# A Practical Guide to Ultrasound-guided Venous Access During Implantation of Pacemakers and Defibrillators

**DOI:** 10.19102/icrm.2022.130204

**Published:** 2022-02-15

**Authors:** Dingxin Qin, Leon M. Ptaszek

**Affiliations:** ^1^Cardiac Arrhythmia Service, Massachusetts General Hospital, Boston, MA, USA

**Keywords:** Axillary vein, cardiovascular implantable electronic device, fluoroscopy reduction, ultrasound guidance, vascular access

## Abstract

Ultrasound (US) guidance has been shown to be a safe and effective option for gaining access to the axillary vein during implantation of cardiovascular implantable electronic devices (CIEDs). However, US-based technique has not been universally adopted in CIED implantations performed in cardiac electrophysiology (EP) laboratories, despite potential advantages over other vascular access techniques. For this reason, not all cardiac electrophysiologists have been trained to use US guidance during CIED implantation. This review is intended to provide a practical guide to the use of US guidance to obtain axillary vein access in the EP laboratory setting.

## Background

The use of ultrasound (US) guidance to access the axillary vein in the context of cardiovascular implantable electronic device (CIED) implantation was first described more than 20 years ago.^1^ Since then, US guidance has been shown to be a safe and effective alternative to other vascular access techniques, including the first rib approach to the subclavian/axillary vein with fluoroscopic guidance^2–4^ and cephalic vein cut-down with direct visualization.^5^

**Figure 1:**
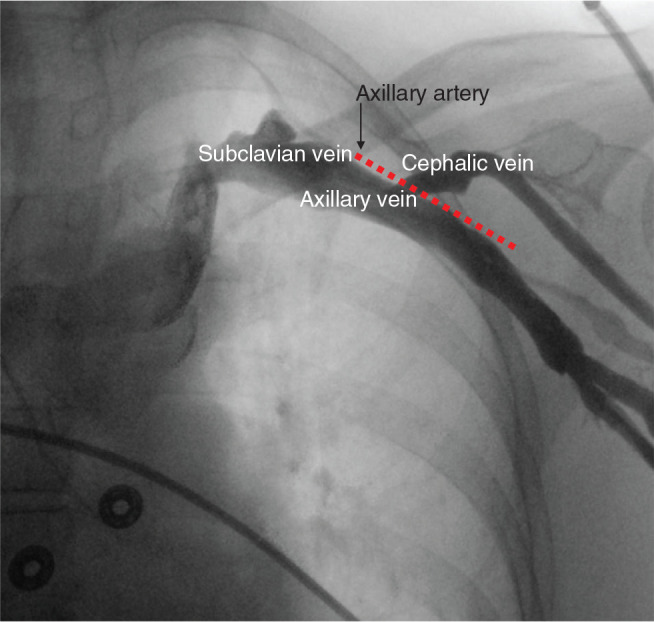
Fluoroscopic evaluation of venous anatomy pertinent to cardiac implantable electronic device implant. Venogram performed with a contrast injection via the left brachiocephalic vein. The locations of the left subclavian, axillary, and cephalic veins are labeled. The approximate location of the left axillary artery is marked by a red dashed line.

US guidance is associated with several potential advantages over other vascular access techniques. US facilitates access to the axillary vein lateral to the costoclavicular junction **(Figure 1)**. A lateral access point may reduce the likelihood of costoclavicular crush injury to lead insulation and conductors compared to a medial access point.^4^ US also allows for the direct visualization of the vein and the needle used for access. This can minimize the risk of pneumothorax associated with a lateral access point, without requiring the use of fluoroscopy.^1,3,5^ US-guided access to the axillary or subclavian vein also requires less tissue dissection than cephalic vein isolation.^4^ This has the potential to reduce tissue trauma and the associated risk of bleeding, particularly in anticoagulated patients. A reduction in bleeding is also likely to reduce the rate of infection.^6^

Despite the advantages associated with US-guided axillary vein access, the technique has not been adopted universally in cardiac electrophysiology (EP) laboratories. For this reason, not all operators in EP laboratories have been trained to use this technique. This review contains a step-by-step description of the procedure and the necessary equipment. Emphasis is placed on aspects of the US-guided access procedure that can maximize the likelihood of successful venous access in challenging situations.^5^

## Description of the ultrasound access technique

### Recommended equipment

Effective use of US guidance for vascular access requires acquisition of high-resolution images in real time. The selected US probe should be sufficiently small to maneuver in a standard-size device pocket. In addition, the probe shape should not impede the maneuvering of the needle during the access procedure. The needle used for vascular access should be as small as possible to minimize the risk of pneumothorax but should be easily visualized with the US probe. The following equipment recommendations are based on experiences reported in the literature.

A probe equipped with a high-frequency, small-footprint linear array transducer (“hockey stick”) has several features that are well suited for this application.^7^ The hockey-stick transducer provides high-resolution imaging at the desired depth (≤6 cm). In addition, the linear array produces a narrow probe footprint, which allows for easier maneuvering of the needle within the pocket. The hockey-stick probe is produced by several different manufacturers and is widely available.

The use of a small-bore vascular access kit (a 21-gauge needle with a 0.018-in guidewire and a 4-French [Fr] sheath with a 3-Fr inner dilator) to obtain vascular access has been described previously.^8^ Obtaining vascular access with a 21-gauge needle can decrease the risk of complications compared to an 18-gauge needle.^9^ Once access is obtained, the 0.018-in guidewire included in the small-bore access kit is exchanged for a 0.035-in, J-tipped guidewire.

### Procedure summary

US-guided access to the axillary vein can be broken down into a sequence of 3 discrete steps. This section contains a description of each step in the procedure for a left-sided implant (the same procedural steps can also be used from the right side). Strategies for avoiding common pitfalls are also provided.

**Figure 2:**
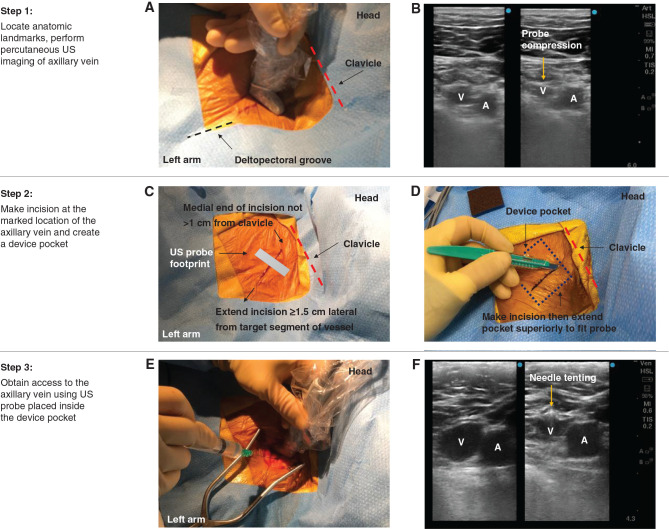
Procedure for ultrasound (US)-guided access to the axillary vein. Step 1: Locate anatomic landmarks and perform percutaneous US imaging of the axillary vein. The locations of major anatomic landmarks are labeled in **A**. The location of the clavicle is marked with a red dashed line, and the location of the deltopectoral groove is marked with a black dashed line. The US probe is placed perpendicular to the skin. The US probe allows for the percutaneous visualization of the left axillary vein (V) and the left axillary artery (A), as shown in **B**. Pressing on the skin with the probe can help distinguish between a vein and artery, as gentle pressure will compress the vein but not the artery. The course of the axillary vein can thus be traced from the clavicle to the deltopectoral groove. The location of the axillary vein can be marked on the skin to guide the incision **(A)**. Step 2: Make an incision at the marked location of the axillary vein and create the device pocket. The marked location of the axillary vein, as determined with percutaneous US **(C)**, is the guide for the incision. The lateral end of the incision should be ≥1.5 from the target segment of the vein to ensure that unobstructed movement of the needle will be possible during access **(C)**. The medial end of the incision should be approximately 1 cm from the clavicle. After the incision is made, the device pocket should be made large enough to accommodate the linear array of the US probe, which will be placed perpendicular to the incision. This may require dissection of the tissue ≤1 cm superior to the incision (dotted line, **D**) in addition to the standard dissection performed inferior to the incision to accommodate the pulse generator. Step 3: Obtain access to the axillary vein using the US probe inside the device pocket. The US probe is covered by a sterile sleeve and then placed in the pocket **(E)**. The probe is oriented so that the axillary vessels are viewed in the short axis **(F)**. The needle is advanced at a 45° angle through the floor of the pocket under direct US guidance **(E)**. Entry of the needle through the anterior wall of the axillary vein is visualized with US **(F)**. The 0.018-in wire is then advanced through the 21-gauge needle.

***Step 1: Locate anatomic landmarks and perform ultrasound imaging of the axillary vein from the skin surface.*** A fluoroscopic view of the vessels utilized for CIED implant is presented in **Figure 1** for reference. To visualize the axillary vein with US, hold the probe perpendicular to the skin just inferior to the clavicle in the mid-clavicular line **(Figure 2A)**. The probe is held perpendicular to the skin surface and is rotated to view the short axis of the vessel. This is frequently referred to as the “out-of-plane” technique.^10^ The axillary vein typically travels inferior and superficial to the artery **(Figures 1 and 2B)**. Compression of the skin overlying the vessels with the US probe can facilitate identification of the vein, as this maneuver will typically lead to the collapse of the lumen of the vein without affecting the artery. Respirophasic collapse of the vein may also be observed without compression of the skin with the US probe. Visualize the vein in multiple locations to define its course from the clavicle to the deltopectoral groove.

***Step 2: Make the incision over the location of the axillary vein, as defined with percutaneous ultrasound imaging, and then create the device pocket.*** Making the incision directly over the target segment of the axillary vein will maximize the ease with which the US probe and the needle are manipulated within the pocket during vascular access. Start by marking the location of the vein, as determined with transcutaneous US, from the clavicle to the deltopectoral groove **(Figure 2C)**. Mark the point on this line at which the vein is best visualized, as this will serve as the initial target for access. Extend the incision ≥1.5 cm lateral to this point, as this will facilitate angulation of the needle lateral to the US probe during vascular access **(Figure 2C)**. The medial end of the incision should be ≤1 cm away from the clavicle.

Administer local anesthetic in the standard fashion around the marked area. Create an incision over the line and extend the incision down to the level of the prepectoral fascia **(Figure 2D)**. Create the device pocket superficial to the prepectoral fascia before attempting access. The long axis of the US transducer will be placed perpendicular to the incision, so the operator should consider extending the pocket 0.5–1 cm superior to the incision to accommodate the probe **(Figure 2D)**. The goal is to have enough room in the pocket so that the vessels will be visible in the center of the US transducer.

***Step 3: Obtain access to the axillary vein using the ultrasound probe in the device pocket.*** Place the hockey-stick US probe within a sterile sleeve, with a small amount (approximately 2 mL) of sterile US gel within the sleeve over the transducer. For imaging inside the pocket, additional US gel does not need to be applied outside of the sleeve. Place the covered US probe on the floor of the pocket and orient the probe to produce a short-axis view of the vessels, as was performed on the skin surface in step 1 **(Figures 2E and 2F)**.

Use a 21-gauge needle to obtain access with direct US visualization. Orient the US probe as close to vertical as possible and rotate to visualize the vessels in the short axis. Compression of the floor of the pocket with the US probe will compress the axillary vein and will help distinguish it from the less compressible artery. Attention should be paid to the approximate depth of the vein to estimate the optimal needle insertion site and angulation. Orient the needle at a 45° angle with respect to the US probe. Insert the needle in the floor of the pocket ≥1 lateral to the US probe **(Figure 2E)**. The insertion point of the needle may need to be adjusted based on the measured distance between the floor of the pocket and the anterior wall of the vein. Distance markers on the US display can assist with this adjustment. Visualize the needle tip and advance it with short, stuttered movements (stuttered advancement of the needle will produce small movements in the surrounding tissue, which will assist in the visualization of the needle tip). Be sure that both the needle tip and the axillary vein are visible in the US view at all times. Achieving this may require small adjustments in the angle of the probe with respect to the floor of the pocket. If the needle tip is not clearly visible with US at any point, remove and re-insert the needle. Access should be through the anterior wall of the vein only. The “through-and-through” approach is not recommended because of the risk of injury to the lung.

Once the needle tip contacts the superficial wall of the vein, compression should be visible on the US **(Figure 2F)**. Once the needle tip is seen in the lumen, aspirate through a syringe to confirm venous blood return. Remove the syringe from the needle without changing the position of the needle tip. If the procedure is being performed by a single operator, it may be necessary to release the US probe to accomplish this (some operators may choose to perform this step of the procedure without a syringe to allow for easier manipulation of the needle). Advance the 0.018-in guidewire through the needle. The guidewire should advance easily, and its position can be confirmed with fluoroscopy. The needle is then removed, and a 4-Fr short sheath with a 3-Fr inner dilator is placed over the guidewire. After the removal of the 0.018-in guidewire and the 3-Fr dilator, a 0.035-inch guidewire can be advanced through the 4-Fr sheath. This allows for the subsequent insertion of a sheath of the desired size.

### Potential procedural pitfalls

***Potential pitfall #1: The axillary vein is not patent.*** The operator should consider the possibility that the vein is not patent before starting the procedure. The likelihood that the vein is not patent is higher in patients with a prior cardiothoracic surgery or who have a previously implanted CIED on the ipsilateral side. In these cases, the operator should consider performing venography **(Figure 1)** to confirm that the vein is patent prior to prepping the site. Percutaneous US mapping of the vein prior to prepping can also be considered.

***Potential pitfall #2: The ultrasound probe is difficult to maneuver inside the device pocket, leading to suboptimal visualization of the axillary vein.*** If the US probe reveals that the axillary vein is at one of the edges of the pocket, expand the pocket in that direction. Repeat US scanning of the vein to confirm that it can be visualized at or near the center of the array. Also, confirm that the US probe can be comfortably rotated within the pocket to maintain a short-axis view of the vessels.

***Potential pitfall #3: The axillary vein is visible with ultrasound, but there is not enough space in the pocket lateral to the ultrasound probe to insert and angulate the needle.*** This is more likely in obese patients in whom the distance between the skin surface and prepectoral fascia is greater. The operator may notice that there is not enough space to maneuver even if the incision was carefully mapped from the skin surface. If space is lacking, extend the incision laterally and expand the pocket laterally until the needle can be inserted and maneuvered without difficulty.

## Discussion

US-guided axillary vein access is a promising technique for improving the outcomes of CIED implantation. Previously published studies have revealed that the US-guided access has a high success rate, low complication rate, shorter procedure time, and low X-ray exposure. In one cohort of 403 consecutive patients who underwent US-guided access for CIED implant by an experienced operator, the rate of successful access was 99%, with no access-related complications.^11^ The average time to obtain venous access was 2.2 minutes. In another case–cohort study comparing 137 cases of US guidance with 679 cases of fluoroscopic guidance or cephalic cut-down for CIED implant,^12^ there was a lower overall complication rate with the US-guided technique, which did not reach statistical significance (2.2% vs. 3.8%; *P* = .36). The use of US was also associated with reduced fluoroscopy time (*P* < .01). In 2018, a randomized trial of US-guided axillary vein access versus conventional subclavian access reported a similar success rate and long-term lead complication rate between the two techniques.^13^ Chandler et al. reported that, in a cohort study of 561 patients, US-guided axillary vein access before incision was safe and could help prevent an extra incision if a barrier is identified that necessitates a change in laterality.^14^

Utilization of in-plane (long-axis) views in US-guided access to the axillary vein has been described.^4^ Although the in-plane technique allows for the visualization of the entire length of the needle, it has been reported that the out-of-plane technique is associated with a higher first-attempt success rate and a shorter procedure duration than the in-plane technique.^10^ This review describes the use of the out-of-plane technique to localize the axillary vein. Obtaining percutaneous, “scout” US images can ensure that the incision and pocket are made directly over the desired access site. This proximity may reduce the challenges imposed by subcutaneous adipose tissue in patients with a large body habitus.^15^

**Figure 3:**
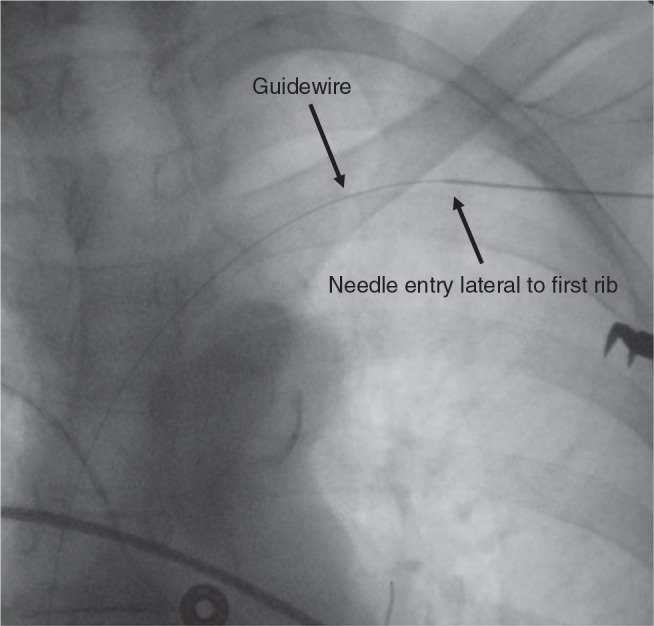
Fluoroscopic view of the point at which the axillary vein is entered using ultrasound (US) guidance. The point at which the tip of a 21-gauge needle was advanced through the anterior wall of the axillary vein with direct US is marked with an arrow. The location of the 0.018-in wire, which was passed through the needle and advanced to the level of the inferior vena cava, is also marked with an arrow.

With the US-guided approach, the venous access site can be more lateral compared to either the anatomic approach to the subclavian vein or the fluoroscopic approach to the axillary vein.^1–5^ The increased distance between the access site and the clavicle may reduce the risk of lead crush injury in the costoclavicular region **(Figure 3)**.

## Conclusions

It has been >20 years since the initial reports of US-guided vascular access for CIED implant.^1,3^ During this period, US-guided vascular access has been shown to be safe and effective in CIED implants. Utilization of a structured approach to US-guided access may help the operator avoid potential procedural pitfalls.
